# Ultrasonographic evaluation of the gallbladder motor function in the diagnosis and prognosis of intrahepatic cholestasis of pregnancy

**DOI:** 10.1186/s12884-023-06209-w

**Published:** 2024-01-02

**Authors:** Minghui Tai, Long Chen, Yajuan He, Fei Wang, Zhen Tian

**Affiliations:** 1 Department of Ultrasound, The First Affiliated Hospital of Xi’an Jiaotong University, No. 277 Yanta Road (w), Xi’an City, Shaanxi province 710061 China; 2 Department of Ultrasound Medicine, Baoji High-Tech Hospital, Baoji, Shaanxi province China; 3 Department of Infectious Diseases, The First Affiliated Hospital of Xi’an Jiaotong University, Xi’an City, Shaanxi province China

## Abstract

**Background:**

Intrahepatic cholestasis of pregnancy (ICP) is characterized by skin pruritus, elevated liver enzymes, and increased serum total bile acids. Several previous studies have revealed that the fasting and ejection volumes of the gallbladder in cholestasis of pregnancy are greater than those in normal pregnancy. The goal of this study was to explore the gallbladder volume and evaluate the diagnostic and prognostic value of ultrasound in ICP.

**Methods:**

We prospectively recruited a cohort of 60 ICP patients at the First Affiliated Hospital of Xi’an Jiaotong University, Shaanxi, China from January 2020 to December 2021 and compared their data with those from healthy pregnant women (n = 60). The gallbladder volume was evaluated by real-time ultrasound examination after overnight fasting and at 30, 60, 120, and 180 min after a liquid test meal of 200 mL, and the ejection fraction was calculated. Continuous data between two groups were compared by Student’s t test. Differences were considered significant for *p* < 0.05. The diagnostic and prognostic value of the volume and ejection function of the gallbladder was analyzed by the receiver operating characteristic (ROC) curve.

**Results:**

The ICP group had significantly higher gallbladder basal volume (43.49 ± 1.34 cm^3^ vs. 26.66 ± 0.83 cm^3^, *p* < 0.01) and higher ejection fraction compared with the healthy group. The ejection fraction higher than 54.55% at 120 min might predict ICP diagnosis with 96.67% sensitivity and 88.33% specificity, and an AUC of 0.9739 (95% CI 0.9521–0.9956), while the gallbladder volume higher than 12.52 cm^3^ at 60 min might predict ICP severity with 59.18% sensitivity and 72.73% specificity, and an AUC of 0.7319 (95% CI 0.5787–0.8852).

**Conclusion:**

Our results indicate abnormal volume and ejection function of the gallbladder in patients with ICP. The ejection fraction at 120 min can assist in the diagnosis if ICP exists, and the gallbladder volume at 60 min may assess the degree of severity of ICP.

**Supplementary Information:**

The online version contains supplementary material available at 10.1186/s12884-023-06209-w.

## Background

Intrahepatic cholestasis of pregnancy (ICP) is a reversible liver condition peculiar to pregnancy. It typically manifests in the late third trimester and is diagnosed in women presenting with classical pruritus associated with elevated liver enzymes and increased serum total bile acids (TBA ≥ 10 µmol/L) [1]. ICP occurs in 1-4% of patients from various nations, and the highest prevalence has been reported in China (5.2%) [2, 3]. ICP is a disorder with multifactorial etiology, including genetic, endocrine, and environmental factors. It is a recognized cause of sudden intrauterine fetal death (IUFD) [4]. Fetal mortality occurs in about 2-4% of ICP pregnancies, and the likelihood of fetal morbidity and stillbirth rises with increasing levels of TBA [5].

Diseases of the liver, biliary system, and intestines are linked to bile acids, which are created from cholesterol in hepatocytes and stored in the gallbladder [6]. The gallbladder is a pear-shaped bag that contains bile. The major functions of the gallbladder include emptying of concentrated bile in response to nutrients, storing hepatic bile during the interprandial period, and mixing of its contents [7]. Although the intrahepatic bile ducts appear normal, some earlier investigations have shown that the gallbladder’s fasting and ejection volumes in pregnancy cholestasis are higher than those in normal pregnancy [8, 9].

Ultrasonographic evaluation of the hepatobiliary system has emerged as one of the most useful techniques for examining liver illnesses because real-time ultrasonography is a cheap, noninvasive, relatively simple, validated, and reproducible technique [10]. Due to its position below the right lobe of the liver and its liquid content, the gallbladder can easily be visualized by ultrasonogcholecystography, which has been recommended as part of the routine ultrasonographic examination of a pregnant woman. Except for our observation, few reports have focused on abnormal volume and ejection function of the gallbladder during ICP. In this study, we further explored the specific ultrasonographic features of the gallbladder and evaluated the diagnostic and prognostic value of ultrasound in ICP.

### Patients

Sixty individuals with ICP were included in the study at the First Affiliated Hospital of Xi’an Jiaotong University, Shaanxi, China from January 2020 to December 2021. All of the participants provided written informed consent, and the study was approved by the Research Ethics Committee of the First Affiliated Hospital of Xi’an Jiaotong University.

No ursodeoxycholic acid was given before the collection of blood samples. The diagnosis of ICP was based on pruritus, elevated liver enzymes, and TBA levels ≥ 10 µmol/L. Sixty healthy pregnant women of similar age with normal serum liver enzymes and TBA levels were recruited as controls. The controls were also in the same gestational age range as the patients with ICP. Preeclampsia, the HELLP (hemolysis, elevated liver enzymes, and low platelets) syndrome, acute fatty liver of pregnancy, primary biliary cirrhosis, viral hepatitis, and any ultrasound abnormality that may result in biliary obstruction were excluded as causes of liver dysfunction.

### Ultrasonographic measurement of the gallbladder volume

**Fig. 1:**
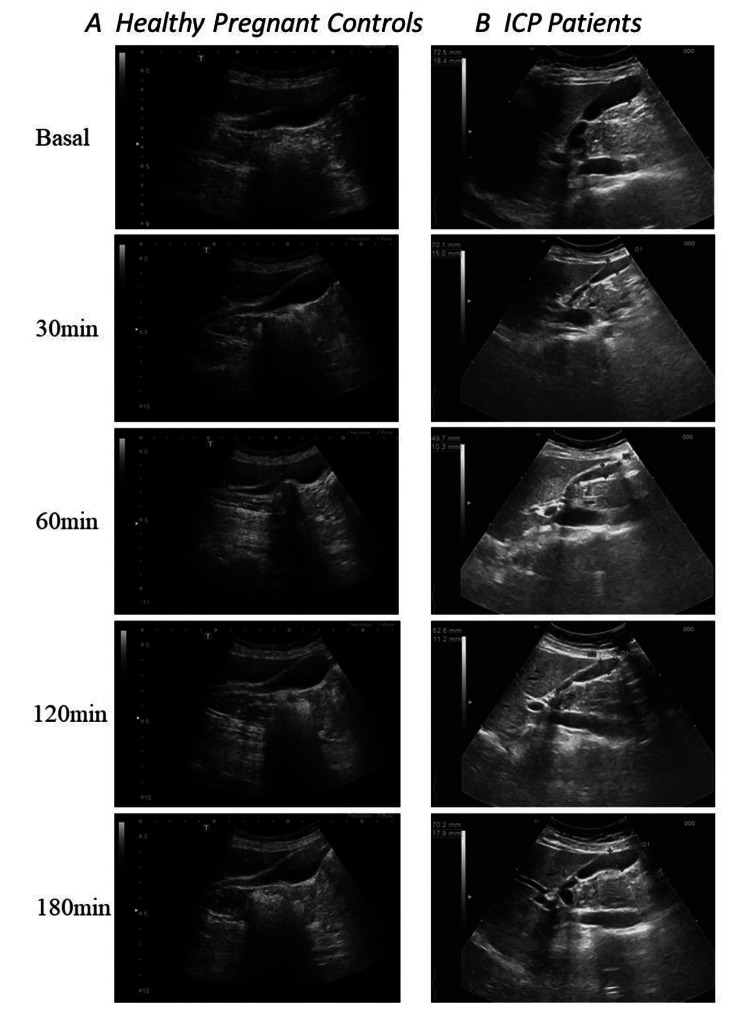
Ultrasonographic measurement of the gallbladder volume. **A**, Ultrasound examination of the gallbladder volume in healthy pregnant women after overnight fasting and 30, 60, 120, and 180 min after a liquid test meal of 200 mL; **B**, Ultrasound examination of the gallbladder volume in ICP patients

The mean gestational period for the ICP group was 32.83 ± 3.23 weeks at the time when ICP was diagnosed, and the mean gestational period for the control group was 32.93 ± 2.07 weeks. The gallbladder volume in the 60 ICP patients and the 60 healthy pregnant women was evaluated by real-time ultrasound examination after overnight fasting for 10 h (from 10:00 p.m. to 8:00 a.m.), and at 30, 60, 120, and 180 min after a liquid test meal of 200 mL [10]. We used a commercially available liquid test meal (Ensure plus, Abbot), which included 200–250 mL liquid solution of about 300 kcal, containing about 40% carbohydrates, 40% fat, and 20% protein, with about 400 mOsm/L. The gallbladder volume was measured and evaluated in real time by two expert operators (MT and LC) using a Philips iU22 system (Philips Ultrasound, Bothell, WA, USA), equipped with a multifrequency convex transducer (C5-2, 5-2 MHz) (Fig. 1A, B). The width, height, and length of the gallbladder were measured. These measurements were used to calculate the gallbladder volume according to the ellipsoid method described by Dodds et al.: Volume of gallbladder [cm^3^] = (0.52 × Length [cm] × Width [cm] × Height [cm]) [11].

### The percentage of gallbladder emptying (ejection fraction)

The percentage of the original volume evacuated by the constriction of the gallbladder is reflected in the ejection fraction (EF), which measures the degree of gallbladder emptying. The percentage of gallbladder emptying was calculated based on the volume differences as follow: [(Fasting gallbladder volume)—(Residual gallbladder volume)] / (Fasting gallbladder volume) × 100% [6].

### Statistical methods

Unless otherwise noted, demographic and clinical data were expressed as mean ± standard deviation (mean ± SD). The Wilcoxon rank-sum test was used to compare continuous variables, while the chi-square test or Fisher’s exact test was used to compare categorical variables. The dynamics of the gallbladder contraction was determined using the regions under the curve. The ability of gallbladder volume and ejection fraction to differentiate the ICP group from the control group was assessed using ROC curves. The maximum of the sum of sensitivity and specificity was used to define cutoffs for continuous variables. The normality of data distribution was assessed using the Kolmogorov–Smirnov test. Data were analyzed using SPSS version 16.0 (IBM Corporation, Somers, NY, USA). A two-tailed *p* value < 0.05 was considered statistically significant.

## Results

### Baseline characteristics

The study cohort included 60 healthy pregnant women and 60 ICP patients. Table 1 displays the clinical baseline characteristics of the ICP patients and healthy controls. The two groups were similar in maternal age, while the ICP group showed significantly higher serum levels of TBA, AST, ALT, ALP, CHOL and gallbladder basal volume. The ICP group demonstrated much earlier delivery than the control group.

**Table 1:** Laboratory parameters in patients with ICP

Characteristics	ICP (n = 60)	HC (n = 60)	*p* Value
Age (yr)	30.68 ± 0.48	29.02 ± 0.63	0.379
Gestational week at delivery (w)	36.77 ± 0.12	37.89 ± 0.12	< 0.01
TBA (µmol/L)	28.11 ± 1.59	7.13 ± 0.22	< 0.01
GB basal volume (cm^3^)	43.49 ± 1.34	26.66 ± 0.83	< 0.01
AST (U/L)	121.15 ± 12.09	30.50 ± 1.83	< 0.01
ALT (U/L)	116.24 ± 12.04	18.75 ± 1.65	< 0.01
ALP (U/L)	110.69 ± 9.07	36.26 ± 2.36	< 0.01
GGT (U/L)	19.42 ± 1.44	18.07 ± 1.39	0.501
CHOL (mmol/L)	6.27 ± 0.25	5.04 ± 0.24	< 0.01
TBIL (µmol/L)	25.16 ± 3.56	20.98 ± 0.98	0.260
DBIL (µmol/L)	15.65 ± 2.51	11.50 ± 0.58	0.130
IDBIL (µmol/L)	9.47 ± 1.20	7.65 ± 0.44	0.156

TBA: total bile acid; AST: aspartate transaminase; ALT: alanine transaminase; ALP: alkaline phosphatase; GGT: gamma glutamyl transferase; CHOL: cholesterol; TBIL: total bilirubin; DBIL: direct bilirubin, IDBIL: indirect bilirubin

### ICP is associated abnormal volume and ejection function of gallbladder

**Fig. 2:**
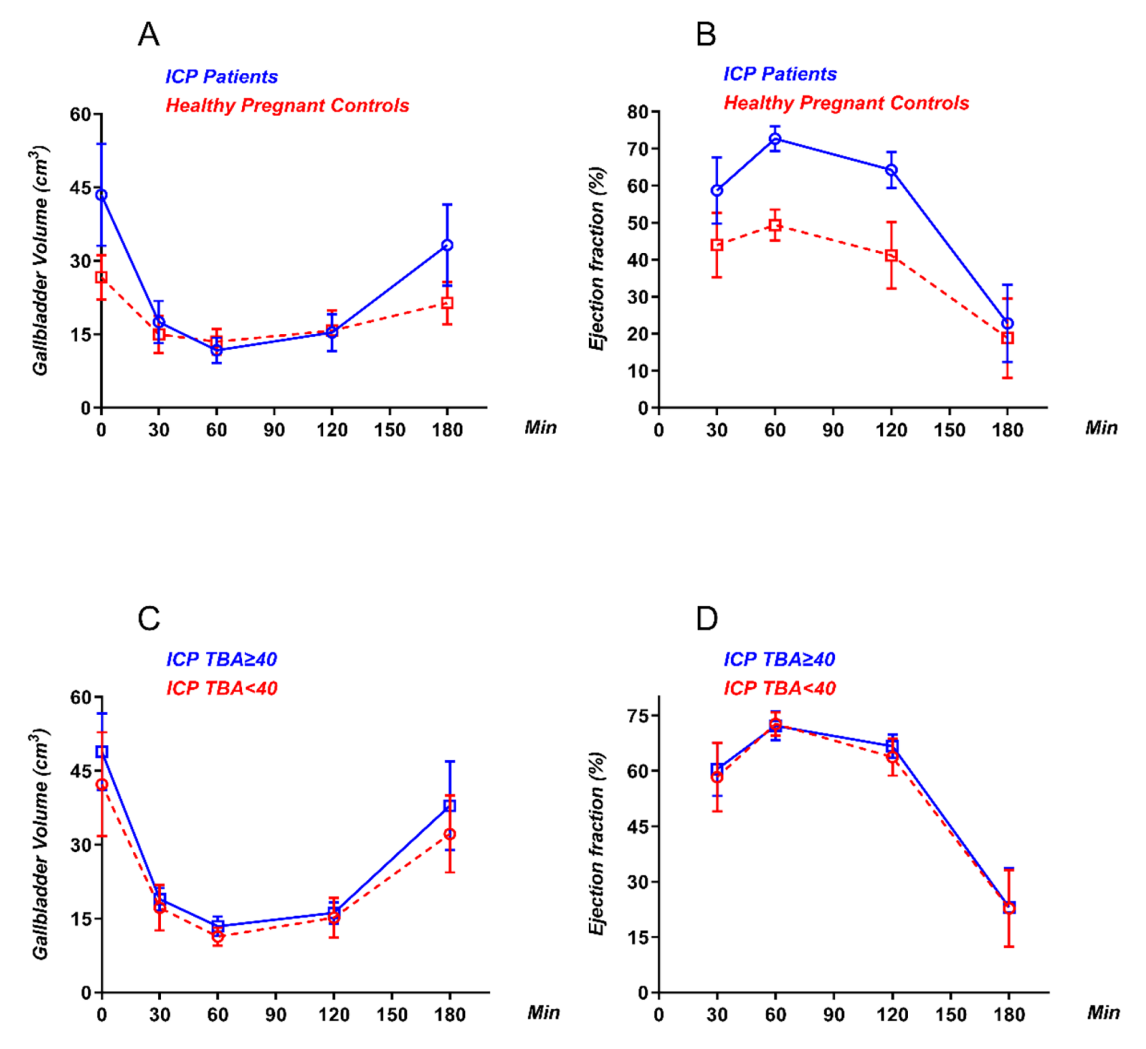
Diagnosis and prognosis of ICP are associated with abnormal volume and ejection function of the gallbladder. **A**, Dynamic changes in the gallbladder volume after overnight fasting and at 30, 60, 120, and 180 min after a liquid test meal; **B**, Dynamic changes in the gallbladder ejection fractions at 30, 60, 120, and 180 min after a liquid test meal; **C**, Dynamic changes in the gallbladder volume after overnight fasting and at 30, 60, 120, and 180 min after a liquid test meal; **D**, Dynamic changes in the gallbladder ejection fraction at 30, 60, 120, and 180 min after a liquid test meal

In both the ICP group and the control group, the maximum contraction was attained after 60 min, and the gallbladder gradually filled to the basal volume over the course of a 3-hour follow-up in all of the pregnant women (Table 2; Fig. 2A, B).

**Table 2:** The result of ultrasonographic gallbladder volume measurement

Parameters	ICP (n = 60)	HC (n = 60)	*p* Value
GB basal volume (cm^3^)	43.49 ± 1.34	26.66 ± 0.83	< 0.01
GB volume at 30 min (cm^3^)	17.52 ± 0.57	14.99 ± 0.70	< 0.01
GB volume at 60 min (cm^3^)	11.72 ± 0.33	13.50 ± 0.48	< 0.01
GB volume at 120 min (cm^3^)	15.38 ± 0.49	15.75 ± 0.76	0.681
GB volume at 180 min (cm^3^)	33.23 ± 1.07	21.38 ± 0.79	< 0.01
GB ejection fraction at 30 min (%)	58.73 ± 1.16	43.96 ± 1.58	< 0.01
GB ejection fraction at 60 min (%)	72.67 ± 0.43	49.37 ± 0.76	< 0.01
GB ejection fraction at 120 min (%)	64.27 ± 0.63	41.19 ± 1.66	< 0.01
GB ejection fraction at 180 min (%)	22.83 ± 1.35	18.86 ± 1.96	0.097

The features of the severe (TBA > 40 µmol/L, n = 11) and moderate (10 < TBA < 40 µmol/L, n = 49) ICP subgroups are shown in Supplementary Table 1 [1]. Maternal age was comparable between the severe and moderate subgroups, while the severe subgroup showed significantly higher serum levels of AST, ALT, CHOL, TBIL, DBIL, IDBIL, and gallbladder basal volume. Three cases of IUFD occurred at 33, 32, and 34 weeks in the severe ICP subgroup.

Compared with the moderate subgroup, the severe ICP subgroup had significantly higher gallbladder basal volume (48.93 ± 2.34 cm^3^ vs. 42.27 ± 1.51 cm^3^, *p* < 0.05). In both the severe and the moderate subgroups, the maximum contraction was attained in 60 min, and the gallbladder gradually filled to the basal volume over the course of a 3-hour follow-up in all of the pregnant women (Table 3; Fig. 2C, D).

**Table 3:** The result of ultrasonographic gallbladder volume measurement (Severity)

Parameters	Moderate ICP (TBA) < 40	Severe ICP (TBA ≥ 40)	*p* Value
	(n = 49)	(n = 11)	
GB basal volume (cm^3^)	42.27 ± 1.51	48.93 ± 2.34	< 0.05
GB volume at 30 min (cm^3^)	17.20 ± 0.66	18.95 ± 0.69	0.229
GB volume at 60 min (cm^3^)	11.33 ± 0.26	13.44 ± 0.59	0.011
GB volume at 120 min (cm^3^)	15.20 ± 0.58	16.16 ± 0.65	0.452
GB volume at 180 min (cm^3^)	32.18 ± 1.12	37.94 ± 2.72	0.037
GB ejection fraction at 30 min (%)	58.34 ± 1.33	60.46 ± 2.19	0.482
GB ejection fraction at 60 min (%)	72.76 ± 0.46	72.25 ± 1.17	0.651
GB ejection fraction at 120 min (%)	63.72 ± 0.72	66.71 ± 0.97	< 0.05
GB ejection fraction at 180 min (%)	22.79 ± 1.49	23.02 ± 3.24	0.947

### The potential diagnostic and prognostic value of the gallbladder volume and ejection fraction for ICP

Using univariate and multivariate Cox regression analyses, we identified the potential risk factors for ICP. As shown in Supplementary Table 2, AST (HR = 1.063, 95% CI: 1.032–1.095, P < 0.01), ALT (HR = 1.072, 95% CI: 1.042–1.102, P < 0.01), ALP (HR = 1.044, 95% CI: 1.027–1.061, P < 0.01), and gallbladder basal volume (HR = 1.219, 95% CI: 1.139–1.304, P < 0.01) were significantly associated with the diagnosis of ICP. We then performed a forward multivariate analysis. The results revealed that gallbladder basal volume (HR = 1.648, 95% CI: 1.047–2.594, P = 0.031) was an independent risk factor for the diagnosis of ICP.

Using univariate Cox regression analyses, we identified the potential value of the volume and ejection function of the gallbladder as a diagnostic and prognostic indicator in ICP (Supplementary Table 3). The gallbladder basal volume, gallbladder volume at 30, 60, and 180 min and gallbladder ejection fraction at 30 and 120 min were significantly associated with the diagnosis of ICP. The gallbladder volume at 60 and 180 min was significantly associated with the severity of ICP.

**Fig. 3:**
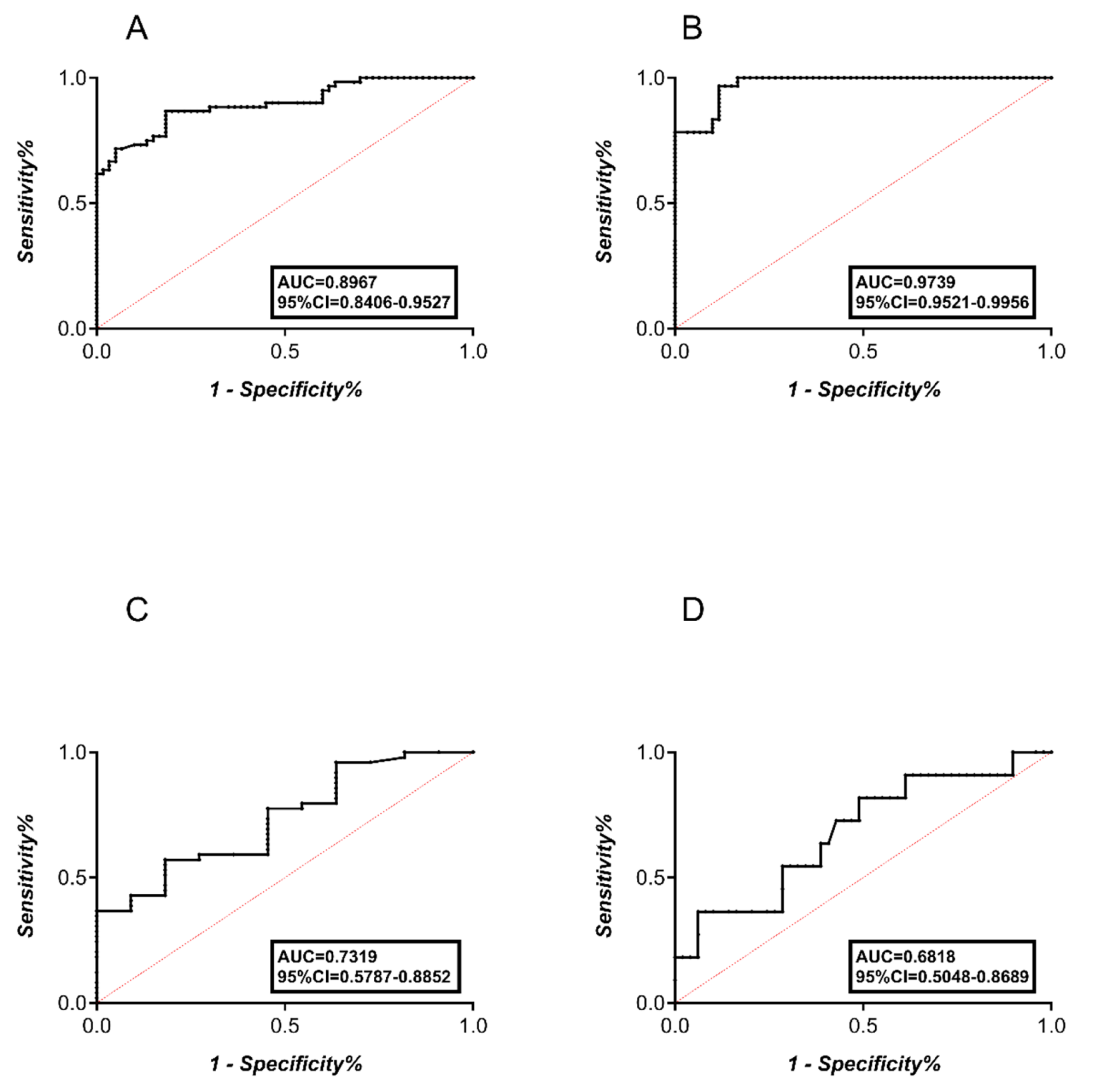
Diagnostic and prognostic value of the volume and ejection fraction for ICP. **A**, **B**, ROC curves for the gallbladder basal volume and the gallbladder ejection fraction at 60 min in the diagnosis of ICP; **C**, **D** ROC curves for the gallbladder volume at 60 and 180 min in the prognosis of ICP

The value of the volume and ejection function of the gallbladder as a diagnostic and prognostic indicator was assessed using the ROC curves (Table 4). The ROC curves of the ejection fraction at 120 min showed a strong separation between the ICP group and the control group, with an area under the curve (AUC) of 0.9739 (95% CI 0.9521–0.9956). The maximum sensitivity and specificity for the ejection fraction at 120 min as the predictor for ICP diagnosis were achieved at 54.55%. The maximum sensitivity and specificity for the gallbladder volume at 60 min as the predictor for ICP severity were achieved at 12.52 cm^3^, with an AUC of 0.7319 (95% CI 0.5787–0.8852) (Fig. 3A-D).

**Table 4:** Diagnostic and prognostic value of volume and ejection fraction for ICP

Parameter	Cut off	AUC	95% CI for AUC		Sensitivity	Specificity	*P*
			Lower	Upper			
**Diagnosis**							
Basal volume (cm^3^)	31.30	0.8967	0.8406	0.9527	81.67	81.67	< 0.01
Volume at 30 min (cm^3^)	15.76	0.6940	0.5982	0.7898	68.33	68.33	< 0.01
Volume at 60 min (cm^3^)	12.62	0.6301	0.5308	0.7294	56.67	56.67	< 0.01
Volume at 180 min (cm^3^)	25.58	0.8636	0.8001	0.9271	76.67	76.67	< 0.01
Ejection fraction at 30 min	51.10	0.8378	0.7667	0.9089	78.33	76.67	< 0.01
Ejection fraction at 120 min	54.55	**0.9739**	0.9521	0.9956	96.67	88.33	< 0.01
**Prognosis**							
Volume at 60 min (cm^3^)	12.52	**0.7319**	0.5787	0.8852	59.18	72.73	< 0.01
Volume at 180 min (cm^3^)	35.55	0.6818	0.5048	0.8689	63.64	61.22	< 0.01

## Discussion

ICP is one of the most common liver diseases closely related to pregnancy. Based on a recently published study, the incidence of ICP in Southwest China is 2.8% [12]. Pregnant women with ICP are at risk of premature delivery, meconium-stained amniotic fluid, intrauterine fetal death, and postpartum maternal hemorrhage [5]. So far, most researchers have made efforts to find serum indicators that could serve as diagnostic and predictive markers in the clinical management of ICP [3, 13, 14]. Although some earlier investigations have shown that the gallbladder’s fasting and ejection volumes in pregnancy cholestasis are higher than those in normal pregnancy [8, 9], no studies have focused on the specific ultrasonographic features of the gallbladder and evaluated the diagnostic and prognostic value of ultrasound in ICP.

Our research showed that the gallbladder volume was increased in ICP patients, and ultrasonographic cholecystographic examination also revealed a sluggish emptying rate of the gallbladder in normal pregnancy. After the test meal, the enlarged gallbladders of the 60 ICP patients were quite capable of contracting. This might be a result of the smooth muscle’s generally higher contraction sensitivity in ICP. Support for this assumption comes from the increased rates of premature births, shorter duration of labor, and stronger uterine contractions in intrahepatic cholestasis of pregnancy than in normal pregnancy [15, 16].

Ultrasonography is the most rapid, accurate and least-invasive diagnostic technique available for assessing diseases of the gallbladder. The assessment of the postprandial percentage of reduction of the gallbladder as a method of evaluating the gallbladder function has already been used in different gallbladder diseases [17–19]. Although the intrahepatic bile ducts appear normal, several earlier investigations have shown that the gallbladder’s fasting and ejection volumes in pregnancy cholestasis are higher than those in normal pregnancy.

The evaluation of the gallbladder motor function by ultrasonography has been used for many years, mostly in patients with gallstone disease, to assess pathological features of the gallbladder function [20, 21]. Gallbladder stasis plays a key role in the pathogenesis of cholesterol gallstones and in conditions associated with gallstone formation, such as vagotomy, pregnancy, use of estroprogestins, total parenteral nutrition, diabetes, and obesity [20]. Gallbladder motility can be examined with “functional” ultrasonography, which helps doctors identify patients with gallstones who can benefit from oral litholysis. Ultrasonography can also be used to evaluate the effect of other medications that may affect gallbladder motility [22].

In the current investigation, we assessed the volume and contractility of the gallbladder in a group of ICP patients and in healthy pregnant women before and after consuming a fatty meal. When the two groups were compared, we found that the baseline gallbladder volume in the ICP patients was significantly higher than that in the healthy controls. A similar finding was obtained when the two groups were examined 180 min after the fatty test meal. At 30, 60, 120, and 180 min after the fatty test meal, ejection fraction differed significantly between the two groups, indicating that the gallbladder volume and ejection fraction might be utilized to diagnose individuals with ICP. The fact that the ejection fraction at 120 min had the greatest AUC of all these markers suggests that it is a more accurate indicator for the diagnosis of ICP. The basal gallbladder volume and gallbladder volume at 60 and 180 min significantly differed between the ICP patients in the severe and moderate subgroups. The ejection fraction differed significantly between the two subgroups of the ICP group at 120 min after the test meal. The fact that the gallbladder volume at 60 min had the greatest AUC of all these markers suggests that it is a more accurate indicator of prognosis in ICP patients.

The current study had some limitations. First, due to the low incidence rate of ICP, the number of ICP patients was limited. In the next step, we will expand the number of ICP patients and conduct multicenter studies to improve the quality of our research. Second, the current study used two-dimensional (2D) rather than three-dimensional (3D) ultrasound. Two-dimensional ultrasound is operator-dependent, and deviations of the gallbladder shape could have affected the results. Compared with 2D ultrasound, 3D ultrasound can accurately measure the gallbladder volume and emptying. Therefore, we will apply 3D ultrasound in our future studies. Third, although there was a statistically significant difference in the gallbladder volume at 60 min between the moderate and severe ICP patients, it appears that the gallbladder volume at 60 min was of limited clinical use for indicating the prognosis of ICP, considering its low sensitivity and specificity. We believe that this was due to a limited number of ICP patients, which resulted in a relative shortage of moderate and severe ICP patients; thus, we will expand the number of ICP patients and conduct multicenter studies in the future.

## Conclusion

We found abnormal volume and ejection function of the gallbladder in patients with ICP. The ejection fraction at 120 min can assist in the diagnosis if ICP exists, and the gallbladder volume at 60 min may assess the degree of severity of ICP. In addition, in the ultrasonographic evaluation of a pregnant woman with liver disease, it is important to keep in mind that a finding of markedly dilated gallbladder does not necessarily indicate extrahepatic obstruction, but it may be the typical and more benign finding due to ICP.

### Electronic supplementary material

Below is the link to the electronic supplementary material.

Supplementary Material 1

## Data Availability

The datasets used and/or analyzed during the current study are available from the corresponding author on reasonable request.

## Abbreviations

ICP: Intrahepatic cholestasis of pregnancy
TBA: total bile acids
IUFD: intrauterine fetal death
UDCA: ursodeoxycholic acid
GB: gallbladder
USG: ultrasonography
EF: ejection fraction
ROC: receiver operating characteristic
AUC: area under the curve
