# Supplementary material for: Ultrasonographic evaluation of the gallbladder motor function in the diagnosis and prognosis of intrahepatic cholestasis of pregnancy
Source: BMC Pregnancy Childbirth. 2024 Jan 2;24:17. doi: 10.1186/s12884-023-06209-w (PMC10759328; doi:10.1186/s12884-023-06209-w)
Supplement: Supplementary file 1 — Supplementary Material 1 [file 12884_2023_6209_MOESM1_ESM.docx]

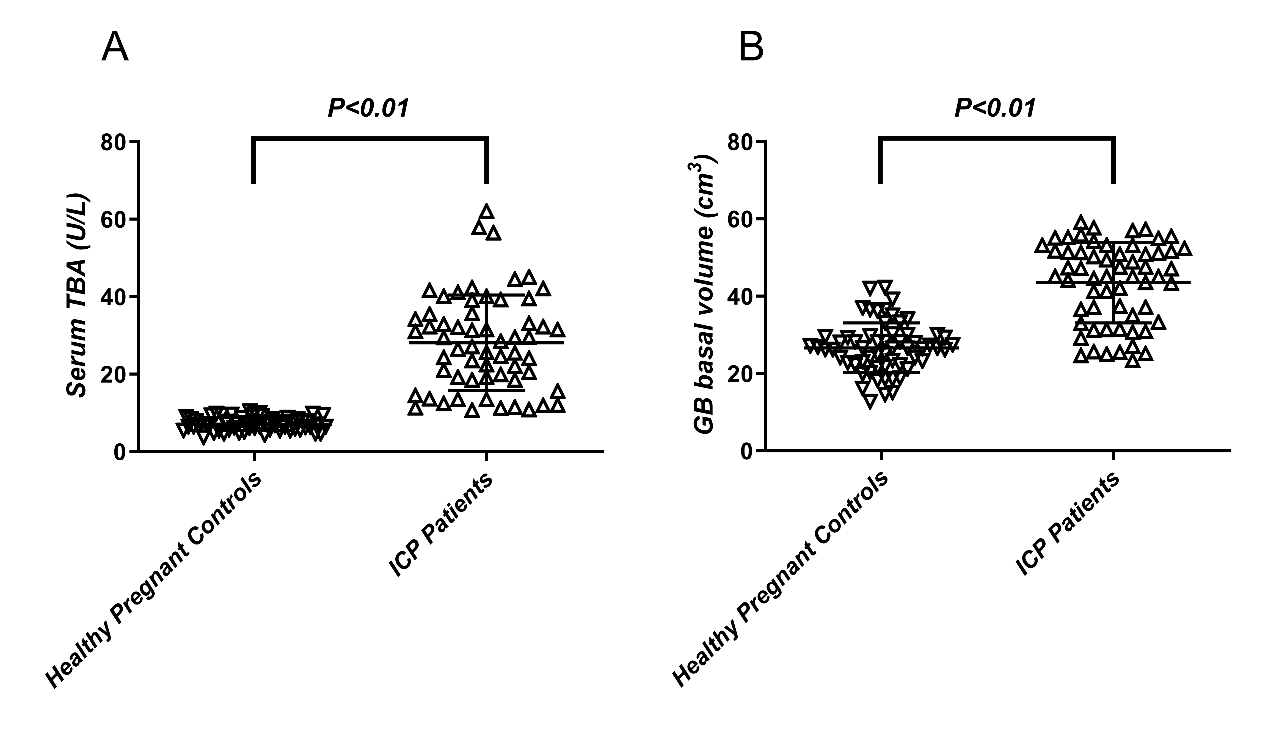


Supplementary Figure 1, TBA levels and Gallbladder basal volumes in healthy pregnant women and ICP patients

A, TBA levels are higher in ICP patients compared with healthy pregnant women;

B, Gallbladder basal volumes are higher in ICP patients compared with healthy pregnant women.

Supplementary Figure 2, Gallbladder basal volumes in severe and moderate ICP patients

Gallbladder basal volumes are higher in severe compared with moderate ICP patients.

Supplementary Table 1 Laboratory parameters in patients with moderate ICP and severe ICP

| Characteristics | Moderate ICP (TBA<40) | Severe ICP (TBA≥40) | *p* Value |
| --- | --- | --- | --- |
|  | (n=49) | (n=11) |  |
| Intrauterine fetal death | 0 | 3 | NA |
| Age (yr) | 30.65 ± 0.48 | 30.82 ± 1.58 | 0.895 |
| Gestational week at diagnosis (w) | 33.14 ± 0.41 | 31.45 ± 1.34 | 0.118 |
| Gestational week at delivery (w) | 36.84 ± 0.13 | 36.38 ± 0.26 | 0.184 |
| Birth weight (g) | 2984.00 ± 55.49 | 2738.25 ± 70.53 | 0.087 |
| GB basal volume (cm^3^) | 42.27 ± 1.51 | 48.93 ± 2.34 | <0.05 |
| AST (U/L) | 89.11 ± 8.76 | 263.87 ± 23.86 | <0.01 |
| ALT (U/L) | 92.83 ± 10.64 | 220.47 ± 30.21 | <0.01 |
| ALP (U/L) | 102.77 ± 8.89 | 145.95 ± 28.43 | 0.065 |
| GGT (U/L) | 18.53 ± 1.64 | 23.36 ± 2.75 | 0.197 |
| CHOL (mmol/L) | 4.75 ± 0.26 | 6.32 ± 0.52 | 0.012 |
| TBIL (μmol/L) | 18.65 ± 1.71 | 54.15 ± 15.53 | <0.01 |
| DBIL (μmol/L) | 11.19 ± 1.14 | 35.53 ± 11.26 | <0.01 |
| IDBIL (μmol/L) | 7.41 ± 0.82 | 18.63 ± 1.66 | <0.01 |

Supplementary Table 2 Uni-and multivariate logistic analysis of risk factors associated with the diagnosis of ICP

|  | Univariate | | | Multivariate | | |
| --- | --- | --- | --- | --- | --- | --- |
|  | HR | 95% CI | *P* | HR | 95% CI | *P* |
| Age (yr) | 1.093 | 1.004-1.190 | 0.060 |  |  |  |
| AST (U/L) | 1.063 | 1.032-1.095 | <0.01 | 1.152 | 0.966-1.375 | 0.116 |
| ALT (U/L) | 1.072 | 1.042-1.102 | <0.01 | 1.192 | 1.005-1.413 | 0.043 |
| ALP (U/L) | 1.044 | 1.027-1.061 | <0.01 | 1.047 | 0.996-1.102 | 0.074 |
| GGT (U/L) | 1.011 | 0.979-1.045 | 0.498 |  |  |  |
| CHOL (mmol/L) | 0.719 | 0.589-0.878 | <0.01 | 0.045 | 0.002-1.235 | 0.066 |
| TBIL (μmol/L) | 1.013 | 0.989-1.037 | 0.295 |  |  |  |
| DBIL (μmol/L) | 1.038 | 0.989-1.089 | 0.131 |  |  |  |
| IDBIL (μmol/L) | 1.043 | 0.982-1.107 | 0.172 |  |  |  |
| GB basal volume (cm^3^) | 1.219 | 1.139-1.304 | <0.01 | 1.648 | 1.047-2.594 | 0.031 |

Supplementary Table 3 Uni-and multivariate logistic analysis of gladder volume associated with the diagnosis & prognosis of ICP

|  | Univariate (diagnosis) | | | Univariate (prognosis) | | |
| --- | --- | --- | --- | --- | --- | --- |
|  | HR | 95% CI | *P* | HR | 95% CI | *P* |
| GB basal volume (cm^3^) | 1.219 | 1.139-1.304 | <0.01 | 1.080 | 0.995-1.172 | 0.067 |
| GB V at 30 min (cm^3^) | 1.115 | 1.029-1.208 | <0.01 | 1.097 | 0.943-1.277 | 0.229 |
| GB V at 60 min (cm^3^) | 0.830 | 0.730-0.945 | <0.01 | 1.512 | 1.065-2.146 | 0.021 |
| GB V at 120 min (cm^3^) | 0.985 | 0.915-1.060 | 0.678 | 1.070 | 0.899-1.274 | 0.446 |
| GB V at 180 min (cm^3^) | 1.231 | 1.145-1.322 | <0.01 | 1.100 | 1.002-1.207 | 0.046 |
| GB EF at 30 min (%) | 1.159 | 1.096-1.227 | <0.01 | 1.029 | 0.950-1.115 | 0.476 |
| GB EF at 60 min (%) | 5.041E+12 | | 0.967 | 0.957 | 0.795-1.153 | 0.645 |
| GB EF at 120 min (%) | 1.399 | 1.231-1.590 | <0.01 | 1.158 | 0.987-1.359 | 0.073 |
| GB EF at 180 min (%) | 1.025 | 0995=1.055 | 0.102 | 1.002 | 0.941-1.067 | 0.946 |
